# Supplementary figures and images for: ELMO1 dependent efferocytosis protects from nephrotoxin induced acute kidney injury
Source: Cell Death Discov. 2026 May 19;12:296. doi: 10.1038/s41420-026-03140-9 (PMC13350932; doi:10.1038/s41420-026-03140-9)

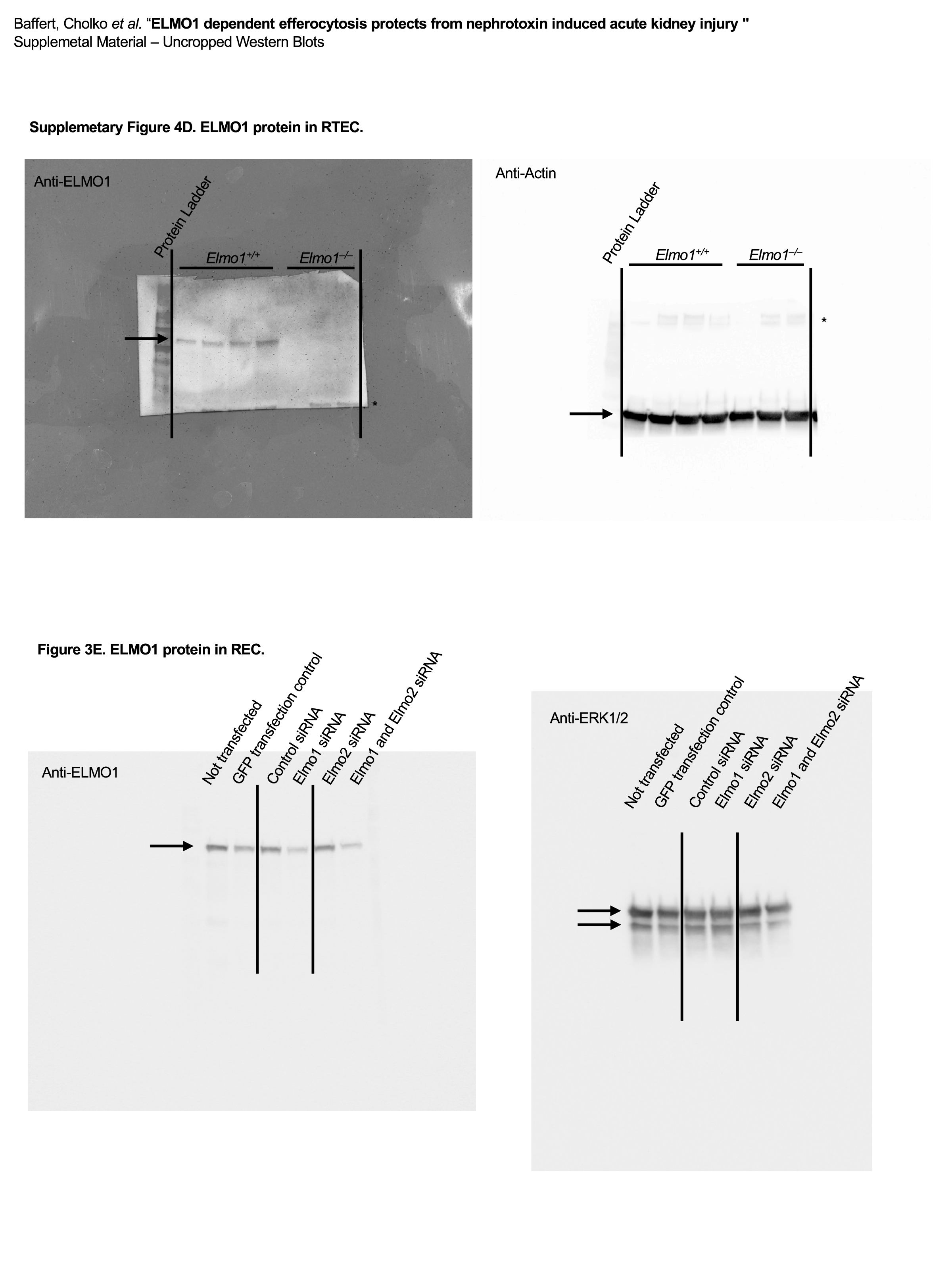

Supplement: Supplementary file 1 — Original Western Blots [file 41420_2026_3140_MOESM1_ESM.png]
