# Supplementary material for: ELMO1 dependent efferocytosis protects from nephrotoxin induced acute kidney injury
Source: Cell Death Discov. 2026 May 19;12:296. doi: 10.1038/s41420-026-03140-9 (PMC13350932; doi:10.1038/s41420-026-03140-9)
Supplement: Supplementary file 2 — Supplementary Material [file 41420_2026_3140_MOESM2_ESM.pdf]

Supplementary Information

ELMO1 dependent efferocytosis protects from nephrotoxin induced acute kidney injury

Blandine Baffert, Michal Cholko, Vikram Sabapathy, Pritika Modhukuru, Isaac Heath, Shuqiu Zheng, Jitendra Gautam, Kevin Schneider, Lily Silverman, Mark Okusa, Rahul Sharma, and Sanja Arandjelovic

Supplementary Figure 1. *Elmo1*$^{-/-}$ mice have normal kidney function at baseline. (**A**) Body weight (g) and kidney weight (g) of healthy *Elmo1*$^{+/+}$ (n=32, 4) and *Elmo1*$^{-/-}$ mice (n=22, 4). (**B**) Serum creatinine and blood urea nitrogen (BUN) levels in healthy *Elmo1*$^{+/+}$ (n=5) and *Elmo1*$^{-/-}$ (n=4) mice. (**C**) Kidney morphology assessed by hematoxylin-eosin (H&E) staining in healthy mice. Representative images are shown. Scale bars indicate 1 mm. Data are shown as mean ± SD; p values were determined by unpaired Student's *t*-test; ns, not significant. Each symbol indicates an individual animal.

Supplementary Figure 1. *Elmo1*$^{-/-}$ mice have normal kidney function at baseline.

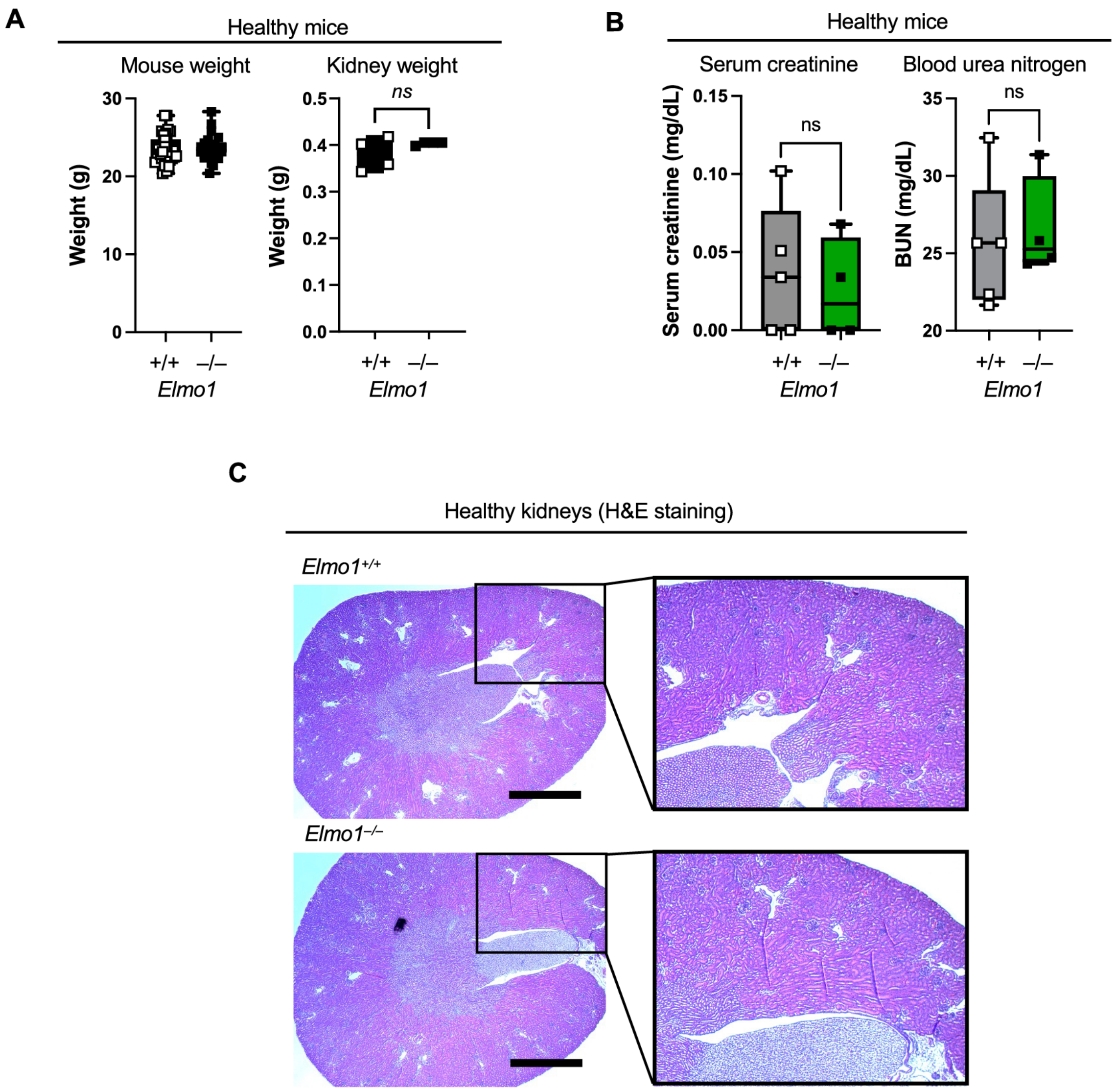

Supplementary Figure 2. ***Elmo1***$^{-/-}$ **mice have reduced inflammatory response after IRI-AKI.** (**A**) Kidney pathology at 24 h post IRI-AKI, assessed by hematoxylin-eosin (H&E) staining. Representative images are shown alongside quantification of kidney pathology scores in *Elmo1*$^{+/+}$ (n=4) and *Elmo1*$^{-/-}$ (n=3) mice. Scale bars indicate 1 mm. Data are from one experiment. (**B**) Relative gene expression of inflammatory markers (*Il-6, Tnfα, Il-10,* and *Il-18*) in kidneys from *Elmo1*$^{+/+}$ (n=11-20) and *Elmo1*$^{-/-}$ (n=7-21) mice following IRI-AKI, normalized to *Hprt1*. Data shown are pooled from more than 3 independent experiments. Each symbol indicates an individual animal. (**C**) Protein levels of IL-6 and TNFα in kidney tissue from *Elmo1*$^{+/+}$ (n=4) and *Elmo1*$^{-/-}$ (n=3) mice on Day 1 after IRI-AKI. Data shown are from one experiment. Each symbol indicated an individual animal. (**D**) Protein levels of IL-6 and TNFα in serum from *Elmo1*$^{+/+}$ (n=4-9) and *Elmo1*$^{-/-}$ (n=3-6) mice following IRI-AKI. Data shown are pooled from two experiments. Each symbol indicated an individual animal. (**E**) Protein levels of neutrophil markers elastase and myeloperoxidase (MPO) in serum from *Elmo1*$^{+/+}$ (n=9) and *Elmo1*$^{-/-}$ (n=6) mice following IRI-AKI. Data shown are pooled from two experiments. Each symbol indicated an individual animal. (**F**) Protein levels of neutrophil markers elastase and MPO in kidney tissue from *Elmo1*$^{+/+}$ (n=4) and *Elmo1*$^{-/-}$ (n=3) mice following IRI-AKI. Data shown are from one experiment. Each symbol indicated an individual animal. (**G**) Representative images of neutrophil infiltration in kidney sections, assessed by Ly6G staining (red), from *Elmo1*$^{+/+}$ (n=3) and *Elmo1*$^{-/-}$ (n=3) mice following IRI-AKI. Nuclei were stained with Hoechst (blue). Data is presented as percentage of total tissue area. Scale bar indicates 1 mm. Data are from one experiment. Data are shown as mean ± SD; p values were determined by unpaired Student's *t*-test; $*p < 0.05$.

Supplementary Figure 2. *Elmo1*$^{-/-}$ mice have reduced inflammatory response after IRI-AKI.

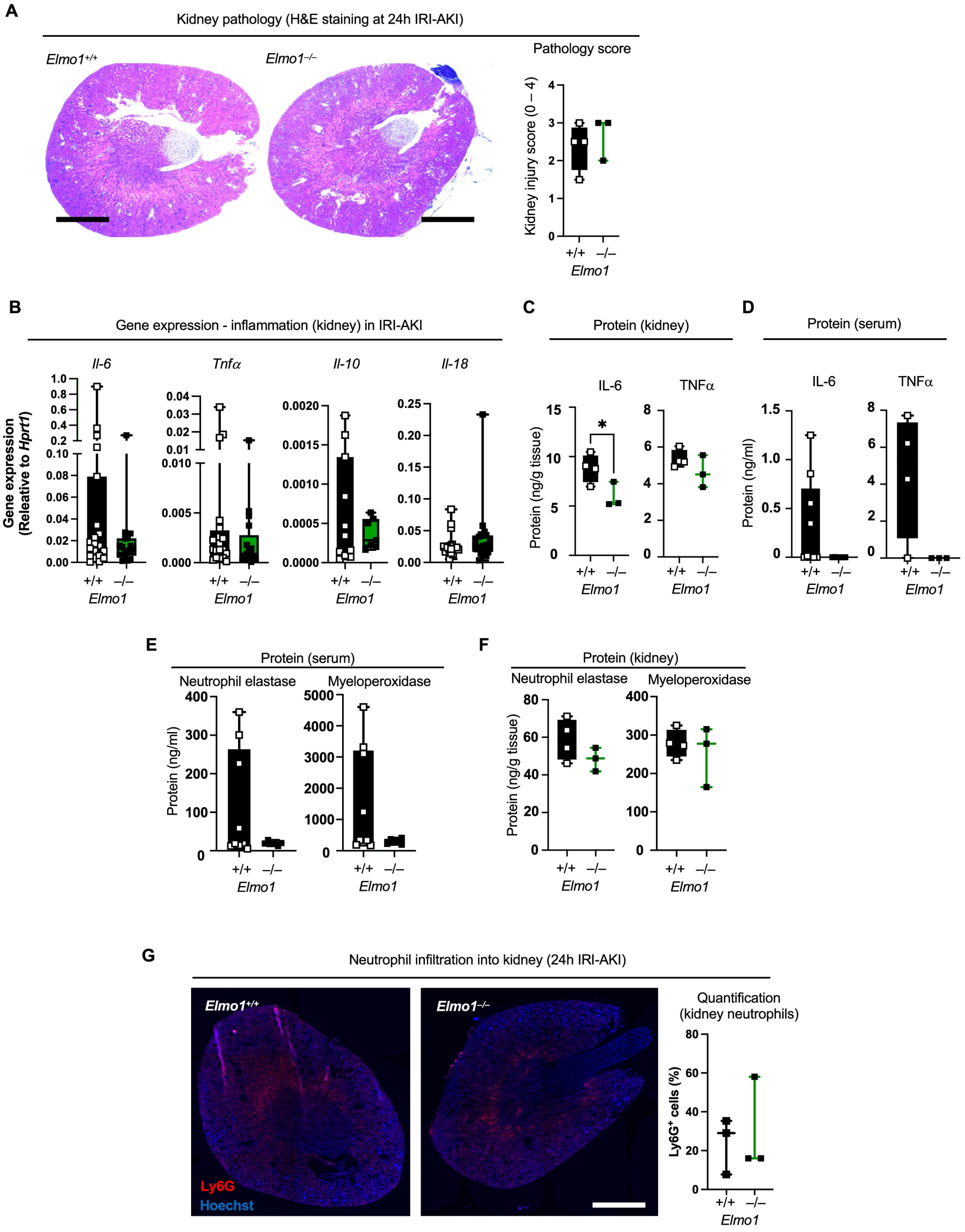

Supplementary Figure 3. *Elmo1$^{-/-}$* kidney inflammatory gene expression in cisplatin-AKI. Relative gene expression of inflammatory markers (*Il-6, Tnfα and Il-10*) in the kidneys from *Elmo1*$^{+/+}$ (n=24), *Elmo1*$^{-/-}$ (n=18), and control$^{+/+}$ (n=5) mice following cisplatin-AKI, normalized to *Hprt1*. Data are pooled from 3 independent experiments. Each symbol indicated an individual animal.

Supplementary Figure 3. *Elmo1*$^{-/-}$ kidney inflammatory gene expression in cisplatin-AKI.

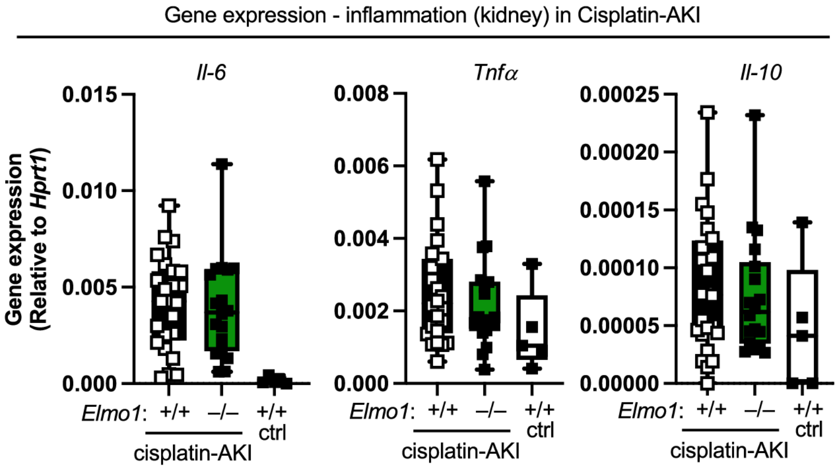

Supplementary Figure 4. Deletion of *Elmo1* does not increase cisplatin-induced cell death in renal tubular epithelial cells (RTEC). (**A**) Flow cytometry gating strategy and purity assessment of cultured primary renal tubular epithelial cells (RTEC). (**B**) Quantification of the percentage of $EpCAM^+$ cells in primary RTEC cultures from $Elmo1^{+/+}$ (n=11) and $Elmo1^{-/-}$ (n=11) mice. Data are pooled from 3 independent experiments. Data are shown as mean ± SEM; p values were determined by unpaired Student's *t*-test. ns, not significant. (**C**) Immunofluorescence staining of cultured primary RTEC for ZO-1 (green) to visualize tight junctions. Hoechst (blue) was used to stain the nuclei. Scale bar, 100 µm. Representative images are shown from one experiment with primary RTEC from $Elmo1^{+/+}$ (n=3) and 3 $Elmo1^{-/-}$ (n=3) mice. (**D**) Immunoblot of protein expression of ELMO1 in cultured RTEC from $Elmo1^{+/+}$ (n=4) and $Elmo1^{-/-}$ (n=3) mice. Actin was used for protein loading control. Each lane represents primary cells established from a separate animal. (**E**) Representative flow cytometry plots of Annexin V and 7AAD stained RTEC from $Elmo1^{+/+}$ (n=3) and $Elmo1^{-/-}$ (n=3) mice after 48 hours of treatment with 125 µM cisplatin. (**F**) Dose-response of cisplatin-induced apoptosis ($AnnexinV^+7AAD^-$) over 48 h in RTEC, with concentrations of 0-125 µM. Graphs show percentage of apoptotic cells. Data are pooled from 2 independent experiments.

Supplementary Figure 4. Deletion of *Elmo1* does not increase cisplatin-induced cell death in renal tubular epithelial cells (RTEC).

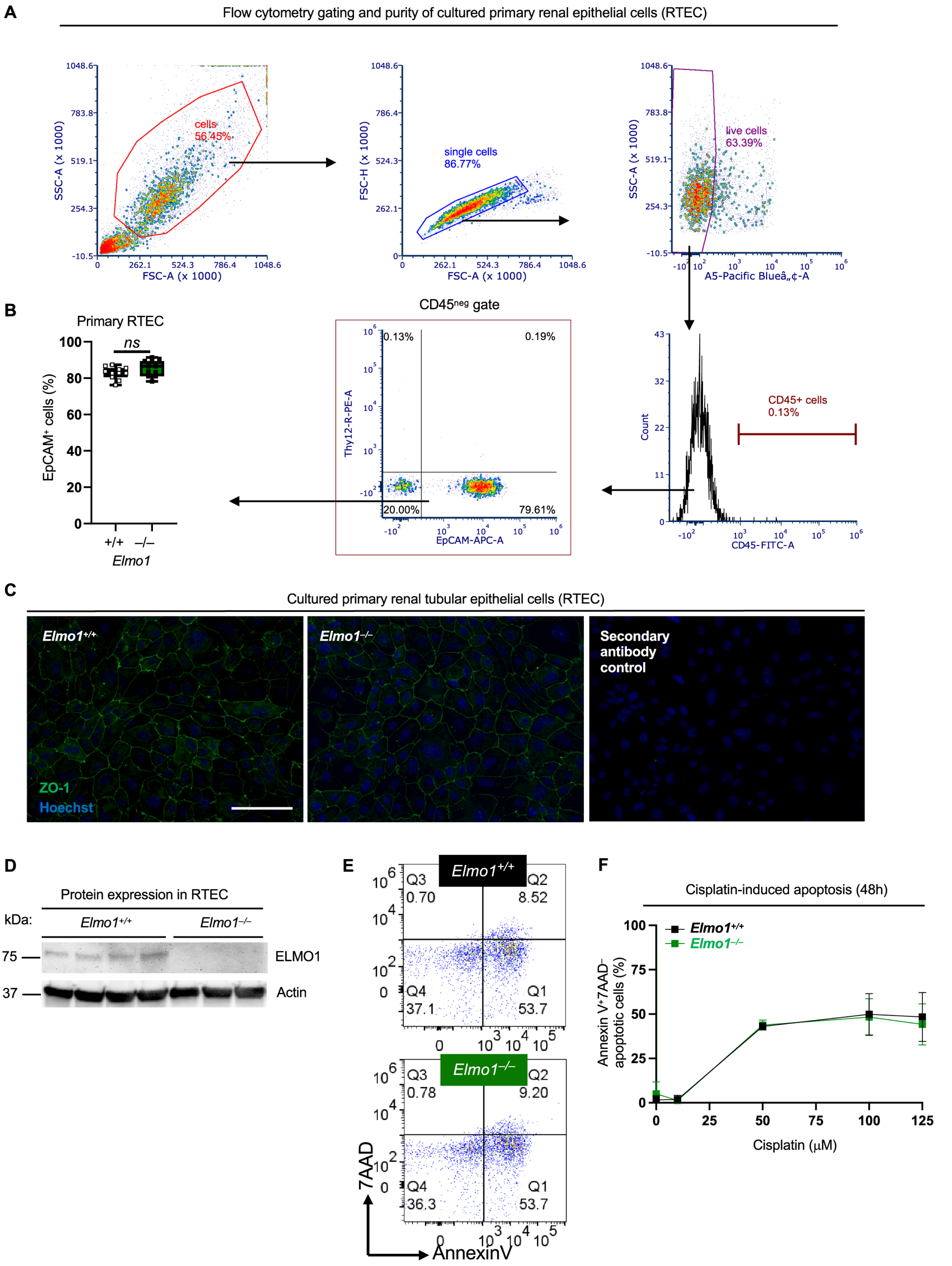

Supplementary Figure 5. Cleaved caspase-3 staining does not overlap with glomeruli in cisplatin-AKI. Representative images of kidney sections from cisplatin-AKI mice stained for cleaved caspase-3. Scale bars indicate 1 mm. Arrows point to glomeruli.

Supplementary Figure 5. Cleaved caspase-3 staining does not overlap with glomeruli in cisplatin-AKI.

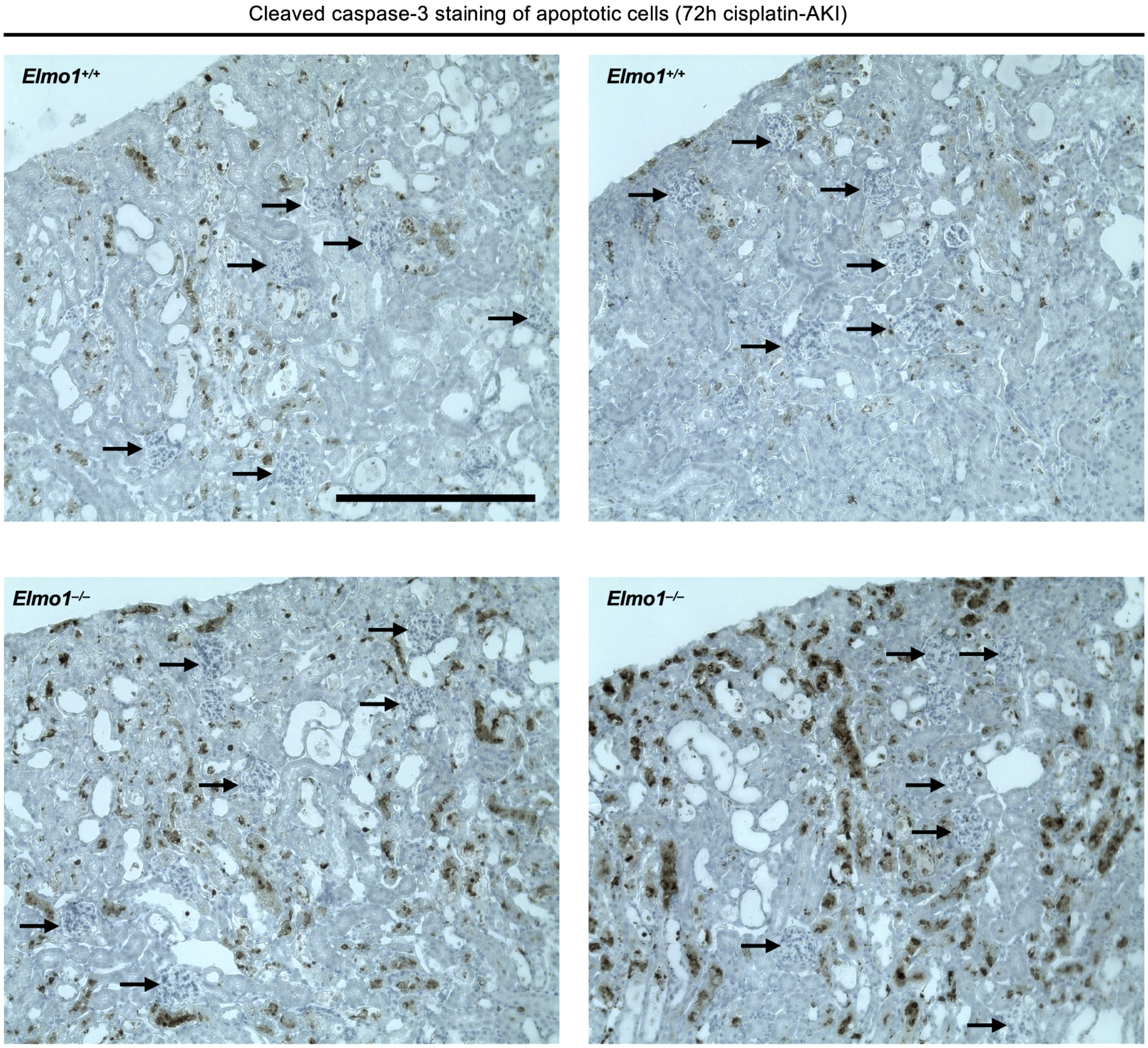

Supplemetary Table 1. List of Databases Used.

**Database Name**	**DOI of Originial Paper**
BioGPS Cell Line Gene Expression Profiles	10.1073/pnas.0400782101
	10.1073/pnas.012025199
	10.1093/nar/gks1114
CTD Gene-Disease Associations	10.1093/nar/gku935
	10.1093/nar/gkn580
DISEASES Experimental Gene-Disease Assocation Evidence Scores	10.1016/j.ymeth.2014.11.020
DISEASES Text-mining Gene-Disease Assocation Evidence Scores	10.1016/j.ymeth.2014.11.020
GAD Gene-Disease Associations	10.1038/ng0504-431
GeneRIF Biological Term Annotations	PMC1480312
GTEx Tissue Gene Expression Profiles	10.1126/science.1262110
	10.1038/ng.2653
GWAS Catalog SNP-Phenotype Associations	10.1093/nar/gkt1229
GWASdb SNP-Disease Associations	10.1093/nar/gkr1182
GWASdb SNP-Phenotype Associations	10.1093/nar/gkr1182
HuGE Navigator Gene-Phenotype Associations	10.1038/ng0208-124
TISSUES Curated Tissue Protein Expression Evidence Scores	10.7717/peerj.1054

**Database Tool**	**DOI**
Harmonizome	10.1093/database/baw100
